# Electronic Screening, Brief Intervention, and Referral to Treatment (e-SBIRT) for Gambling Harm: A Mixed-methods Acceptability Study

**DOI:** 10.1007/s10899-025-10424-9

**Published:** 2025-09-05

**Authors:** Simon Wright, Jessica Smith, Glen Dighton, Martyn Quigley, Simon Dymond

**Affiliations:** 1 School of Psychology, Swansea University, Singleton Campus, Swansea, SA2 8PP UK; 2 Department of Psychology, Reykjavík University, Reykjavik, Iceland

**Keywords:** E-SBIRT, Brief intervention, Gambling, Acceptability, Mixed-methods

## Abstract

Gambling harm is a significant public health burden, yet treatment uptake is low. Electronic screening, brief intervention, and referral to treatment (e-SBIRT) programmes have potential to increase uptake and improve treatment outcomes. However, no studies to date have investigated e-SBIRT in the context of gambling. We conducted a single-arm mixed-methods study of acceptability of e-SBIRT for gambling. Quantitative acceptability was indicated by users’ perceived satisfaction, impact and helpfulness of the e-SBIRT. Qualitative acceptability was explored using semi-structured interviews. Participants (*n* = *63*), pre-screened for gambling severity, reported high levels of satisfaction with the e-SBIRT, found it helpful, and were more likely to seek treatment. Participants with higher gambling severity scores found the e-SBIRT more acceptable and were more likely to seek treatment following the intervention. Qualitative feedback (*n* = 7) supported the e-SBIRT’s acceptability. The present findings support the acceptability of e-SBIRT for gambling. Further research is required to refine the intervention and examine its effectiveness with those with gambling harm.

**Supplementary Information:**

The online version contains supplementary material available at 10.1007/s10899-025-10424-9.

## Introduction

Gambling disorder (GD) is defined as a persistent pattern of gambling despite experiencing substantial distress or impairment (American Psychiatric Association, 2013). A range of harms have been identified relating to gambling, including detrimental effects to financial stability, physical and psychological health, relationships, employment and education (Public Health England, 2021; Wardle et al., 2019). Within Great Britain, for instance, the prevalence of problem gambling, a commonly used term to describe individuals who gamble in a manner that creates problems across multiple domains (Tran et al., 2024), has been reported to be 2.5% (Gambling Commission, 2024). Despite the array of potential gambling harms, only one in five experiencing harms are estimated to seek treatment (Bijker et al., 2022).

Screening, brief intervention, and referral to treatment (SBIRT) is a public health approach to reduce harm associated with addictive disorders (Babor et al., 2007). To do this, SBIRT programmes involve screening for harmful behaviours, followed by the provision of either feedback and education regarding risk, brief intervention, and/or referral to treatment depending on the severity of the harm (Babor et al., 2007). SBIRT programmes have been found to be effective in the reduction of harm caused by substance misuse (Baker et al., 2005; Barata et al., 2017; Bernstein et al., 2005), and recently, have been applied in the context of gambling. Heinlein et al. (2022) conducted a single arm pilot feasibility study of a gambling specific SBIRT in a HIV/primary care clinic. In this study, 15 participants received the SBIRT delivered by three clinicians, and measures of preliminary effectiveness were gathered at baseline, post-intervention and 1-month follow-up. It was found that participants reduced the number of days gambled as well as the median amount of money spent on gambling. Nehlin et al. (2016) investigated a SBIRT-style intervention within primary care health services. In their study, patients were screened, and those experiencing harm from gambling were offered a return visit to speak about their gambling habits. Of the 537 screened, 34 were invited back, yet only six completed the return visit at 1-month follow-up. Taken together, these results suggest that SBIRT programmes have the potential to reduce harm from gambling but need to be designed to reduce attrition.

Owing to technological advances, client-accessed electronic-SBIRT (e-SBIRT) programmes have gained popularity in recent years. Jones et al. (2024) conducted a systematic review and meta-analysis of e-SBIRT for addictive disorders, concluding that e-SBIRT has the potential to offer the same benefits of in-person SBIRT, whilst being less demanding in terms of cost and time. A range of studies have shown that e-SBIRT programmes are effective in reducing harm caused by substance misuse. Jo et al. (2019) evaluated the efficacy of an e-SBIRT programme designed to change risky drinking behaviour. In this study, 1496 participants were randomised to the intervention or a control involving assessment with the provision of generic feedback. Participants in the e-SBIRT group consumed less alcohol in the past week, displayed less binge drinking behaviour and had overall lower alcohol use scores. Other studies have examined e-SBIRT in the context of alcohol as well. Rubin et al. (2022) investigated the impact of an e-SBIRT delivered via a computer agent on hazardous drinking rates and referrals to specialty care. In this study, 178 participants were randomised to an e-SBIRT group or e-SBIRT + treatment as usual. Assessments were administered at baseline and 3-month follow-up. It was reported that whilst both groups decreased their drinking, the e-SBIRT group evidenced greater improvements in alcohol related consequences and were significantly more likely to receive a brief intervention and referral to speciality care. Collectively, the above studies demonstrate preliminary evidence in favour of e-SBIRT for the treatment of substance misuse.

Gambling disorder and substance use frequently occur together (Yarbakhsh et al., 2023) and share several characteristics, including a range of psychological and physical health problems (Karlsson & Håkansson, 2018; Roerecke & Rehm, 2013; Strang et al., 2020; Wejbera et al., 2021) and low reported levels of treatment seeking (Bijker et al., 2022; Fuller et al., 2022). Given the evidence in favour of e-SBIRT programmes for substance use, e-SBIRT may be a promising approach to the reduction of harm from gambling. Moreover, given that e-SBIRT allows users access to support at a time and place that is favourable to them, it has the potential to reduce the attrition observed with in-person SBIRT (Nehlin et al., 2016). However, no studies to date have investigated the potential acceptability of e-SBIRT to reduce harm caused by gambling.

To address this gap, the present study sought to establish the acceptability of an e-SBIRT programme for gambling. Quantitative acceptability was defined by users’ perceived satisfaction, impact and helpfulness of the e-SBIRT. Qualitative acceptability was indicated by participants’ positive and negative comments about the e-SBIRT and whether users were more likely to seek treatment following completion of the intervention. A further aim was to examine the impact of gambling severity on acceptability.

## Methods

### Participants & Procedure

Participants were recruited from Prolific and directed to a survey hosted on Qualtrics. Recruitment took place over two phases between December 2024 and January 2025. In phase one, participants completed the Problem Gambling Severity Index (PGSI; Ferris & Wynne, 2001). Participants were eligible for inclusion if they were aged 18 or above, resided in the UK and had gambled in the past month. In phase two, participants who scored in the moderate or problem gambling categories of the PGSI were recontacted using Prolific IDs and invited to take part in the main study. After obtaining written consent, participants were informed they would view a programme designed to help people with their gambling. Participants then completed the e-SBIRT and a range of acceptability measures. The e-SBIRT was completed in one session and took approximately 10 min. All participants were invited to take part in semi-structured interviews evaluating the e-SBIRT. Among those that agreed, participants were separated by gambling severity category (moderate vs problem gambling) and selected randomly to ensure an even representation of each category was obtained. There were no significant differences between those that completed the interviews and the full sample. The average interview length was 18 min (range = 13–26).

### Design

This study was a single-arm mixed-methods design. Acceptability measures are listed below and please see Supplementary Materials for a copy of the interview schedule. Ethical approval was obtained from Swansea University Human Research Ethics Committee (2 2024 11481 10694).

### Acceptability Measures

#### Satisfaction

Satisfaction with the e-SBIRT was assessed using the 3-item Client Satisfaction Questionnaire (CSQ-3; Attkisson & Greenfield, 1995). Each item of the CSQ-3 is rated on a 4-point Likert scale. Total score ranges between 3 and 12, with higher scores indicating greater overall satisfaction, participant needs met and likelihood of future participant use.

#### Impact

Impact of the e-SBIRT on participants’ awareness, knowledge, attitude, intention to change, treatment-seeking behaviour and behaviour change in relation to gambling was assessed using the 6-item App-Specific subscale of the Mobile App Rating Scale (MARS). To ensure the questions were relevant, we substituted the word ‘app’ for ‘survey’ on each item, which were rated on a 5-point Likert scale ranging from 1 (strongly disagree) to 5 (strongly agree). The MARS has demonstrated excellent inter-rater reliability and internal consistency ((Cronbach’s α = 0.90); Stoyanov et al., 2015).

#### Perceived Helpfulness

The perceived helpfulness of each component was assessed using a single item rated on an 11-point scale ranging from 0 (not at all helpful) to 10 (very helpful).

#### Treatment Seeking Behaviour

Treatment seeking behaviour was assessed using the single item ‘I am more likely to seek support for my gambling having completed this survey’ where participants responded on a 5-point Likert scale ranging from 1 (strongly agree) to 5 (strongly disagree).

### e-SBIRT

**Fig. 1:**
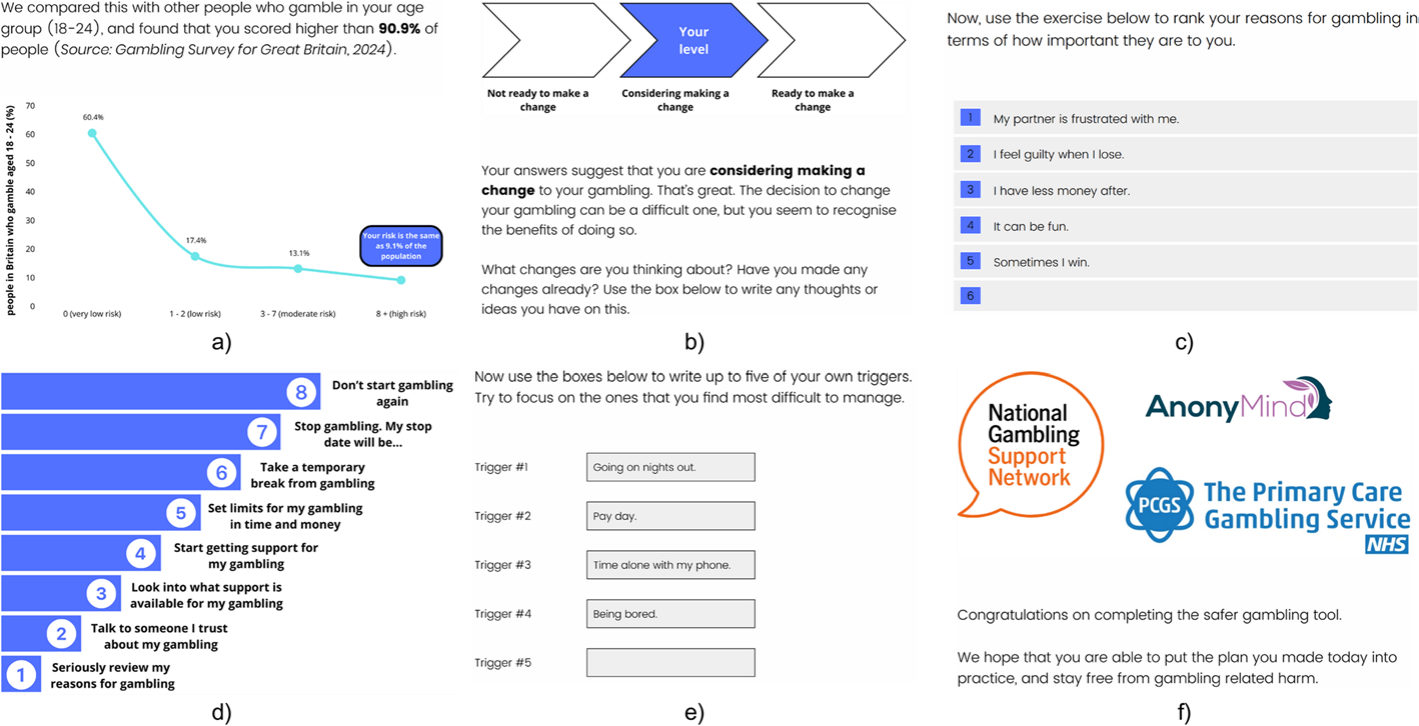
Example screenshots of the e-SBIRT; **a** Normative feedback and psychoeducation, **b** Readiness to change and feedback, **c** Decisional balance, **d** Goal setting, **e** Relapse prevention, **f** Referral to treatment

The e-SBIRT was based on principles of motivational interviewing (MI) (Miller & Rollnick, 1991; Yakovenko et al., 2015) and included a range of behaviour change techniques. A summary of the e-SBIRT is detailed below and additional screenshots can be seen in Fig. 1. The e-SBIRT was completed using Qualtrics and users did not receive any external input. An anonymous copy can be accessed at https://swanseachhs.eu.qualtrics.com/jfe/form/SV_74AsfSfgQwoCoZ0.

#### Screening

Participants were screened using the PGSI (Ferris & Wynne, 2001). The PGSI assesses past-year gambling severity and contains 9 items rated on a 4-point scale (0 never to 3 almost always). Participant’s scores can be placed into four categories: non-problem (score of 0), low risk (score of 1–2), moderate risk (score of 3–7) and high risk ‘problem gambling’ (score of 8 or higher). In our sample, the PGSI demonstrated excellent internal reliability (α = 0.88).

#### Brief Intervention

The brief intervention was tailored to the users’ gambling severity. Depending on participant responses, the brief intervention was comprised of normative feedback on gambling (e.g., “We compared this with other people who gamble in your age group [18–24], and found that you scored higher than 90.9% of people”), psychoeducation on the consequences of gambling, an assessment of readiness to change with feedback (e.g., “Your answers suggest that you are considering making a change to your gambling right now”), a decisional balance exercise, a goal setting exercise and a relapse prevention exercise (e.g., “Now it’s time to create a plan for how to cope with these triggers without gambling”). Additional information can be found in the Supplementary Materials.

#### Referral to Treatment

After completing the brief intervention, participants received a message congratulating them on completing the e-SBIRT and encouraging them to seek support from a range of agencies. The listed agencies included the National Gambling Support Network, the Primary Care Gambling Service and the NHS website for gambling addiction.

### Analysis

Quantitative data was analysed using JASP (version 0.19.3). Acceptability was explored using descriptive statistics. Differences between PGSI categories was examined using Mann–Whitney U tests due to non-normality of data. An alpha level of 0.05 was adopted for all analyses. Qualitative data from the interviews was analysed using inductive thematic analysis (Braun & Clarke, 2006). This involved a five-phase process of (1) familiarising with the data, (2) generating initial codes, (3) searching for themes, (4) reviewing themes and (5) defining and naming themes. Coding was independently carried out by two authors (SW and JS), and then an iterative process of cross-examining coding strategies and data interpretation was performed to establish a consensus. Following initial coding, similar responses were inductively analysed to generate content themes. Final themes were verified by an independent author (GD).

## Results

### Quantitative

#### Sample & Demographics

A total of 445 participants completed phase one of recruitment. Of these, 179 were invited to take part in the main study, of which 72 participated. Data from nine participants was excluded due to PGSI scores outside the moderate or problem gambling categories (*n* = *8*) and participants not passing the attention check (*n* = *1*). Therefore, the final sample was 63. The mean age was 39 years (*SD* = *13.40*), 39 (61.90%) were male, 44 (69.84%) reported their ethnicity as White and 34 (57.14%) stated they were in full-time employment. A total of 34 (53.97%) met the PGSI criteria for moderate risk gambling, with the remaining 29 (46.03%) with scores indicative of problem gambling (*mean PGSI* = *8.68; SD* = *5.51*).

#### Acceptability

Descriptive statistics for the satisfaction, impact and perceived helpfulness of the e-SBIRT are shown in Table 1.

**Table 1:** Descriptive statistics for acceptability

Acceptability measure	Value, mean (SD)	95% CI
Satisfaction (1–4; CSQ-3)		
The intervention met my needs	3.16 (0.77)	2.97–3.35
Overall satisfaction with the intervention	3.51 (0.69)	3.33–3.68
I would use the intervention again, if needed	3.33 (0.65)	3.17–3.50
Total satisfaction with intervention	10.00 (0.72)	9.69–10.31
Impact (1–5; MARS)		
Awareness of gambling harm	4.30 (0.82)	4.01–4.51
Understanding of gambling harm	4.33 (0.76)	4.14–4.53
Attitudes towards gambling harm	4.13 (0.79)	3.93–4.33
Motivation to address gambling harm	4.27 (0.79)	4.07–4.47
Help seeking for gambling harm	4.08 (0.79)	3.88–4.29
Reduction in harm from gambling	3.84 (1.05)	3.58–4.11
Perceived helpfulness (0—10)		
Normative feedback and psychoeducation	7.71 (1.96)	7.22–8.21
Readiness to change and feedback	7.81 (2.00)	7.31–8.31
Decisional balance	7.64 (2.32)	7.05–8.22
Goal setting	8.00 (2.13)	7.46–8.54
Relapse prevention	7.84 (1.93)	7.36–8.33

*SD* standard deviation, *95% CI* 95% confidence interval, *CSQ-3* Client Satisfaction Questionnaire, *MARS* Mobile app rating scale

##### Satisfaction

On the CSQ-3, participants scored a mean of 10.00 out of 12 (*SD* = 0.72), indicating overall satisfaction with the e-SBIRT. With respect to individual items, most participants reported that the e-SBIRT met most of their needs, that they were very satisfied with the e-SBIRT, and that they would likely use the e-SBIRT again to help with their gambling.

##### Impact

The mean scores across the MARS ranged from 3.84–4.33 (*M* = 4.16) indicating the e-SBIRT met acceptability standards (minimum score of 3) (Creber et al., 2016). From highest to lowest impact, participants reported that the e-SBIRT improved their understanding of gambling, awareness of gambling, motivation to address gambling, attitude toward gambling, likelihood of seeking treatment for gambling and reduction of harm from gambling having completed the e-SBIRT.

##### Perceived Helpfulness

The mean ratings for the perceived helpfulness of the e-SBIRT components were all greater than 5 out of 10, indicating above-average helpfulness. The component rated as most helpful was the goal setting exercise (*M* = 8.00, *SD* = 2.13). The component rated as least helpful was the decisional balance exercise (*M* = 7.64, *SD* = 2.32).

##### Treatment Seeking Behaviour

When asked if participants were more likely to seek treatment following completion of the e-SBIRT, the majority (52.38%) agreed.

#### Impact of Gambling Severity on Acceptability

Analyses were conducted to determine if acceptability differed between PGSI categories. In terms of satisfaction, those with scores indicative of problem gambling (*Mdn* = 4.00, *IQR* = 1.00, *M* = 3.45) were significantly more satisfied with the e-SBIRT than those with scores indicating moderate risk of problem gambling (*Mdn* = 3.00, *IQR* = 1.00, *M* = 3.23, *U* = 3711.00, *p* = 0.033, rank biserial correlation [*rrb*] = 0.16). For impact, problem gambling scores (*Mdn* = 4.00, *IQR* = 1.00, *M* = 4.34) lead to significantly higher ratings of the impact of the e-SBIRT than scores indicative of moderate risk (*Mdn* = 4.00, *IQR* = 1.00, *M* = 4.01, *U* = 14,258.50, *p* < 0.001, *rrb* = 0.20). With respect to perceived helpfulness, those with scores indicating problem gambling (*Mdn* = 9.00, *IQR* = 3.00, *M* = 8.17) perceived the e-SBIRT as significantly more helpful than those with moderate risk scores (*Mdn* = 8.00, *IQR* = 3.00, *M* = 7.49, *U* = 9463.00, *p* < 0.001, *rrb* = 0.23). Finally, participants with scores suggesting problem gambling status (*Mdn* = 4.00, *IQR* = 1.00, *M* = 4.14) were significantly more likely to seek treatment following completion of the e-SBIRT than those at moderate risk (*Mdn* = 4.00, *IQR* = 1.00, *M* = 3.59, *U* = 307.50, *p* = 0.005, *rrb* = 0.38).

### Qualitative

#### Sample & Demographics

A total of 42 participants agreed to complete semi-structured interviews, of which seven took part. Of these, five were male (71.42%) and four (57.14%) met the PGSI criteria for moderate risk gambling with the remaining three meeting criteria for problem gambling (*mean PGSI* = *9; SD* = *5.32*).

#### Interview Data

**Fig. 2:**
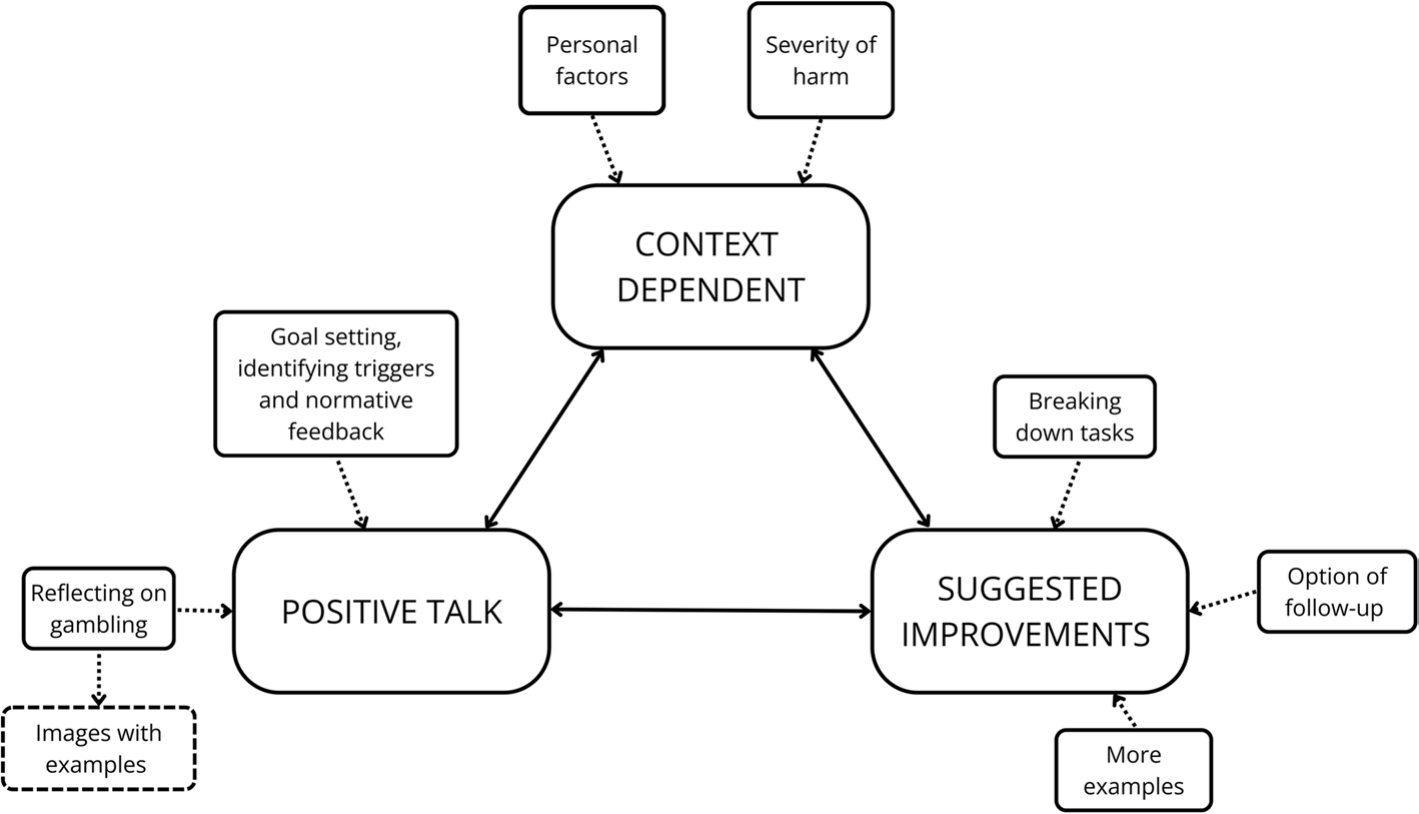
Thematic map; overview of themes and subthemes

Participants’ comments were reflected in three overarching themes: (1) Positive talk, (2) Suggested improvements and (3) Context dependent (see Fig. 2). In this analysis, all themes were interlinked; many of the suggested improvements involved expanding the aspects users liked. Likewise, participants’ personal circumstances and context was found to shape their perceptions of the e-SBIRT.

#### Theme 1 – Positive Talk

Most comments were positive and broadly fell into two sub-themes: (1) Reflecting on gambling (subtheme: Images with examples) and (2) Goal setting, identifying triggers and normative feedback.

##### Reflecting on Gambling

Most participants remarked that completing the e-SBIRT caused them to reflect on their gambling. Extending this further, one participant commented that the e-SBIRT caused them to re-evaluate their current gambling as potentially problematic: “*I never really saw it as a problem. I thought I was quite casual, but answering a survey highlighted that, oh, okay, maybe I did have a problem*” [P4, female].

##### Images with Examples

Participants indicated that the use of images helped them reflect on their gambling behaviour. One participant expanded on this, describing how images with examples helped them think about their own gambling: “*What I really liked is where you had the kind of bubble diagram for want of a better word when you had lots of different box bubbles, colours, different sizes with some key headings in there, it helps you with food for thought.*” [P3, male].

##### Goal Setting, Identifying Triggers and Normative Feedback

Across all participants, the goal setting, identifying triggers and normative feedback aspects received praise and were considered their own sub-themes. Four participants indicated that they found the goal setting exercise helpful, commenting that the act of writing down goals gave them motivation. Four participants said they found identifying their triggers helpful. For example: “*I like especially the trigger thing like thinking about what actually makes you gamble is something I never thought about before. Like what scenarios or what emotions, I think boredom is one of the main things for me*.” [P2, male]. Three participants mentioned they found receiving normative feedback on their gambling helpful, with one individual adding that the graphs were particularly motivating.

#### Theme 2 – Suggested Improvements

Several participants suggested improvements which were organised into three sub-themes: (1) More examples, (2) Breaking down tasks and (3) Option of follow-up.

##### More Examples

Three participants commented that they would find more examples helpful. Specifically, one person suggested using more examples in the trigger identifying exercise, whilst another suggested examples could be incorporated in the form of case studies.

##### Breaking Down Tasks

Two participants expressed that the option to break down tasks would be helpful. This was suggested for the goal setting exercise, as well as the section on consequences from gambling, where they suggested breaking these down by severity.

##### Option of Follow-up

Participants generally commented that the option of a follow-up would be helpful, rather than using the e-SBIRT on a single occasion. For example: “*The really important bit about setting specific goals and then chasing up on those could be really helpful for some people.*” [P7, male].

#### Theme 3 – Context Dependent

Participants expressed that their perceptions were shaped by contextual factors, which fell into two sub-themes: (1) Severity of harm and (2) Personal factors.

##### Severity of Harm

A common theme expressed by participants was that the e-SBIRT would be more helpful for those with ‘less severe’ gambling problems. For example: “*I suppose it depends what level your gambling's got to. If it's of a low level, I can see something like this being really helpful to get you back on track. Personally, I think if it was really high level and it was sort of affecting other areas of your life, I'm not 100% sure how helpful this survey would be*.” [P1, male].

##### Personal Factors

Participants expressed that a range of personal factors shaped their perception of the e-SBIRT. For example, one participant highlighted the importance of where an individual was in their gambling harm journey: “*I suppose it depends what stage you're at. If you're at the stage when you're not ready to admit…you might just ignore it*” [P1, male].

## Discussion

The aims of this study were to examine the acceptability of an e-SBIRT programme for gambling for the first time and to investigate the impact of gambling severity. Overall, the e-SBIRT was rated favourably across all measures. Participants indicated that they were satisfied with the e-SBIRT, that it was impactful with respect to their gambling, and that they found all components helpful. We also noted an impact of gambling severity, with greater acceptability among those who scored in the problem gambling category on the PGSI.

The finding that participants found the e-SBIRT helpful is consistent with Yakovenko et al.’s (2015) systematic review and meta-analysis of MI which concluded that it is an efficacious method in the treatment of GD. Whilst our intervention was partly based on MI, it may be prudent to consider other modalities and interventions. Hawker et al. (2021) examined the acceptability of an app-delivered intervention to reduce cravings to gamble. Two components rated most helpful were distraction and a mindfulness practice to ‘surf’ the wave of a craving to gamble without acting on it. Future iterations of the e-SBIRT should consider such components, as well as MI-related components from other interventions (Forman et al., 2025).

We found broad alignment between the quantitative measures and the interview feedback. However, one area which did not align was the impact of gambling severity on the perceived helpfulness of the e-SBIRT. Specifically, participants who scored in the problem gambling category rated their perceived helpfulness higher than those in the moderate risk category. In contrast, during the interviews participants expressed that the e-SBIRT would be more helpful for those with ‘less severe’ problems. One explanation for this may relate to the time window captured by the PGSI, which reflects past-year gambling (Ferris & Wynne, 2001). Therefore, the PGSI scores obtained may reflect historical gambling behaviours which have subsequently changed. Relatedly, the view that the e-SBIRT alone would be insufficient for those with more severe gambling problems is consistent with the SBIRT model (Babor et al., 2007) which recommends individuals at greater risk are facilitated into an appropriate treatment structure.

A theme which emerged from the interview data was that participants found the goal setting, identifying triggers and normative feedback components of the e-SBIRT especially useful. These findings partially align with a recent Delphi study where clinicians ranked relapse prevention and goal setting as the 1 st and 3rd most effective change techniques in gambling treatment respectively (Keshani et al., 2025). Interestingly, although normative feedback was cited as one of the most helpful elements of our e-SBIRT, social comparison received the lowest rank in the Delphi study (Keshani et al., 2025). Future research examining the effectiveness of social comparison approaches will help elucidate the impact of this technique. A second theme which emerged from the interview data was that participants found examples useful, with one user suggesting the incorporation of case studies. The use of case studies has been demonstrated in other e-SBIRT programmes in the context of substance misuse (Boudreaux et al., 2013; Haskins et al., 2017) and may represent an area of development for our e-SBIRT.

Importantly, most participants indicated that completing the e-SBIRT would result in them being more likely to seek treatment. Given that only 20% of those experiencing harm from gambling seek treatment (Bijker et al., 2022), this is of particular relevance. One explanation for this is that digital interventions may reduce stigma, which is a commonly reported barrier to seeking treatment for gambling (Gainsbury et al., 2014). Additionally, this approach may help ameliorate the comparatively high rates of attrition observed with in-person SBIRT (Nehlin et al., 2016), leading to greater reductions in harm from gambling. Future studies that incorporate a longitudinal design should seek to verify whether users do indeed seek treatment following engagement with an e-SBIRT.

A key advantage of e-SBIRT is that it has the potential to offer the same benefits of in-person approaches, whilst being less demanding in terms of cost and time (Jones et al., 2024). Within the UK, an individual seeking treatment for gambling typically undergoes assessment and screening, followed by a tier 2 referral which involves a brief intervention underpinned by Cognitive Behavioural Therapy and MI delivered by public or third sector providers (Seel et al., 2024). This may represent an ideal space to consider the use of e-SBIRT; indeed, the latest UK guidance on gambling related harms (National Institute for Health and Care Excellence [NICE], 2025) stipulate that MI should be considered to improve people’s commitment to change and that interventions should include relapse prevention (NICE, 2025). Given that our e-SBIRT includes such elements, it may be a suitable intervention within the UK treatment sphere.

The findings of this study should be interpreted with respect to several limitations. One limitation was that lower risk categories (PGSI < 2) were not considered and therefore results cannot be generalised to this population. However, the brief intervention and referral to treatment aspects of e-SBIRT are typically delivered to users who display at least a moderate level of risk and/or harm (Babor et al., 2007). It was therefore appropriate to select a sample whose severity of gambling was moderate or higher. A second limitation was that we did not recruit participants who were the target population for the e-SBIRT. Typically, e-SBIRTs are intended for treatment-seeking populations. It was therefore not possible to explore all aspects of our intervention among its intended population of use. To overcome this, future research should focus on treatment-seeking populations.

## Supplementary Information

Below is the link to the electronic supplementary material.

Supplementary file1 (PDF 113 KB)

## Data Availability

Quantitative data is available at https://osf.io/f9pqk/. Qualitative data is available upon request.
