# Supplementary material for: Electronic Screening, Brief Intervention, and Referral to Treatment (e-SBIRT) for Gambling Harm: A Mixed-methods Acceptability Study
Source: J Gambl Stud. 2025 Sep 5;41(4):1583–96. doi: 10.1007/s10899-025-10424-9 (PMC12657564; doi:10.1007/s10899-025-10424-9)
Supplement: Supplementary file 1 — Supplementary file1 (PDF 113 KB) [file 10899_2025_10424_MOESM1_ESM.pdf]

Electronic screening, brief intervention, and referral to treatment (e-SBIRT) for gambling harm: A mixed-methods acceptability study

Journal of Gambling Studies

Simon Wright[1], Jess Smith[1], Glen Dighton[1], Martyn Quigley[1], and Simon Dymond[1,2].

[1]School of Psychology, Swansea University, United Kingdom.

[2]Department of Psychology, Reykjavík University, Iceland.

Corresponding author(s)

Simon Wright, School of Psychology, Swansea University, Singleton Campus, Swansea SA2 8PP, UK. ORCID iD: Simon Wright 0009-0001-0868-8298. Email: 2380375@swansea.ac.uk

Supplementary Materials

Supplementary Material 1.1 – Semi Structured Interview Schedule

Please note that prompts such as 'can you tell me a bit more about that?' may have been used throughout.

1) What did you think of the survey?

2) Did you like or dislike any parts of the survey in particular?

3) Do you think anything was missing from the survey? What would you like to see added?

4) Do you think anything was not needed in the survey?

5) Are there any changes that you would make to the survey in general?

6) What did you think to the language used in the survey, would you change anything about it?

7) What did you think to the length of the survey?

8) What did you think to the overall design of the survey, is there anything that you would change?

9) The aims of the survey were to identify people at risk of gambling harm, provide a brief intervention and motivate people to seek support. In its current form, do you think the survey does that?

10) Are there any barriers to the survey in terms of achieving the aims I just mentioned, if so, what are they and how could we overcome these?

11) In your opinion, what do you think the most important aspect that needs to be changed or improved is?

Supplementary Material 1.2 – Brief intervention: Additional Information

<u>Normative feedback on gambling</u>: Normative feedback was provided based on the participants PGSI score and age. If participants scored in the non-problem or low-risk categories, they received age-based normative feedback with an affirming message and viewed tips on how to maintain safe gambling before being directed out of the survey. If participants scored in the moderate or high-risk categories, they received age-based normative feedback, a supportive message and were directed to the next section of the e-SBIRT. Data used in the normative feedback was taken from the Gambling Survey for Great Britain (Wardle et al., 2023).

<u>Psychoeducation on the consequences of gambling:</u> Participants were provided with a statement detailing the frequency of individuals that have experienced negative consequences due to their gambling in the past year. Participants were then presented an infographic of the common negative consequences arising from gambling. Data used in this section was taken from the Gambling Survey for Great Britain (Wardle et al., 2023).

<u>Assessment of readiness to change with feedback</u>: Readiness to change was assessed using the Gambling Readiness to Change Questionnaire (GRTC) (Neighbors, 2002). The GRTC is adapted from the Alcohol Readiness To Change Questionnaire (Rollnick et al., 1992) and is a 9-item scale with three items measuring each of the three stages: precontemplation, contemplation and action. In this, participants indicate the extent to which they agree with each item, from 1 (strongly disagree) to 5 (strongly agree). The GRTC has demonstrated satisfactory reliability for the composite scale (alpha = 0.81) and each of the three subscales, with alphas of 0.64, 0.80 and 0.74 for precontemplation, contemplation and action respectively (Neighbors, 2002). After completion of the GRTC, participants received feedback on their current stage of change alongside an affirming message. Consistent with Miller and Rollnick (1991), participants that asserted they were in the pre-contemplation or contemplation stage were directed to a decisional balance exercise designed to increase motivation for behaviour change. Participants that asserted they were in the action stage were directed to a goal setting exercise, as they had already acknowledged a need for behaviour change (Neighbors, 2002).

<u>Decisional balance exercise</u>: Participants were informed the exercise was designed to help with their motivation for change by reviewing their personal relationship with gambling. Participants were then instructed to write up to three positive and negative things about their gambling, before ranking them in terms of how important they are to them. Participants were encouraged to reflect on their motivation for change having completed the exercise.

<u>Goal setting exercise</u>: This exercise was adapted from the HERA exercise 'Stairway to Change - where are you?' (Boudreaux et al., 2013). Participants viewed an image with some example goals relating to gambling and

were instructed to reflect on their own goals. Participants then completed up to three of their own goals before completing fields on how and when they were going to complete these.

Relapse prevention exercise: This exercise was adapted from the HERA exercise ‘Breaking the chains’ (Boudreaux et al., 2013). In this, participants were provided with information on gambling triggers and viewed an image with some example triggers. They were then instructed to write up to five of their own triggers and then write a plan on how to manage these without engaging in gambling.
